# Root growth and yield responses to nitrogen levels in bell pepper (*Capsicum annuum*) cultivation: balancing nutrient efficiency and productivity

**DOI:** 10.3389/fpls.2025.1589560

**Published:** 2025-09-08

**Authors:** Sharon Chemweno, Daniel Owusu Kwakye, Shimon Rachmilevitch, Jhonathan E. Ephrath, Naftali Lazarovitch

**Affiliations:** ^1^ The Albert Katz International School for Desert Studies, The Jacob Blaustein Institutes for Desert Research, Ben-Gurion University of the Negev, Sede Boqer, Israel; ^2^ French Associates Institute for Agriculture and Biotechnology of Drylands, The Jacob Blaustein Institutes for Desert Research, Ben-Gurion University of the Negev, Sede Boqer, Israel

**Keywords:** root phenotyping, minirhizotron, total root length, root length density, fertigation

## Abstract

**Introduction:**

The root system plays a key role in plant nutrient and water uptake, influencing growth, yield, and nitrogen use efficiency (NUE). However, excessive N fertilizer is often applied to boost productivity but can instead reduce efficiency and cause nitrate leaching, leading to underground water pollution. Optimizing N fertilization requires an understanding of root system response to nitrogen.

**Methods:**

We examined the effect of different N rates on root growth using *in situ* minirhizotron (MR) and destructive root study methods (ingrowth core, soil core, and soil excavation). In a net-house experiment, bell peppers (*Capsicum annuum*) were examined under different N concentrations: 100 ppm (control), 50 ppm (moderate-N), and 25 ppm (low-N).

**Results:**

Reduced N concentrations reduced leaf gas exchange and shoot biomass, but promoted root growth. Across all four methods, root length density (RLD), total root length (TRL), and root surface area significantly increased under reduced N. RLD values in the upper 30 cm of the soil profile were significantly higher under the low and moderate-N treatments compared to the control treatment, while fine roots (<2 mm thickness) exhibited increased RLD with low-N treatment. MR system recorded a higher RLD of ~70% and 33% compared to ingrowth core and excavation, respectively, likely due to loss of fine roots during washing.

**Discussion:**

Our findings indicate that while reduced N application significantly enhanced root growth, resource allocation varied between low and moderate-N treatments. The moderate-N treatment achieved a balance, supporting both increased root development and yield. In contrast, the low-N treatment enhanced root growth and NUE but did not translate into higher yield. This suggests that N-induced root system plasticity is critical in optimizing nutrient uptake efficiency and ensuring balanced resource allocation for both root and shoot development, as demonstrated by the moderate-N treatment.

## 1 Introduction

Nitrogen is the most essential element for plant growth, accounting for 1%–5% of total dry matter and ~80% of nutrients absorbed by plants (Marschner, 2011). In 2021, ~109 Tg of N fertilizer was used globally (FAO, 2023), representing an increase of ~30 kg ha^–1^ of cropland over the preceding year. This usage is expected to increase to 150 Tg yr^–1^ by 2050. However, crop nitrogen use efficiency (NUE) ranges globally from 30% to 50% of total applied N, with a low NUE of ~14% reported for vegetables grown in greenhouses and net-houses (Cassman et al., 2002; Zhang et al., 2015). With a low NUE, excess applied N is lost to the environment, with direct economic losses in agriculture (Andersen et al., 1999; Garnett et al., 2009) and pollution issues such as eutrophication, high NO_3_^–^ concentrations in groundwater, and N_2_O emissions (Zhu et al., 2005).

Bell pepper (*Capsicum annum* L.) is a vegetable crop grown extensively worldwide, mainly in greenhouses and net-houses, especially in the Mediterranean region, with health benefits as a source of vitamins A and C and a role in nutritionally balanced diets (Sezen et al., 2019). Such vegetable production is often associated with N fertilizer application that exceeds crop demand. This vegetable has a low NUE, meaning that substantial amounts of N are lost, resulting in negative environmental effects (Gomez-Lopez and Del Amor, 2013; Padilla et al., 2018; Thompson et al., 2020). Previous studies have shown that excessive N application to vegetables such as bell peppers has negative effects such as increased vegetative growth with reduced yield productivity and quality (Bar-Tal et al., 2001; Stefanelli et al., 2010; Yasuor et al., 2013). The notable yield reduction with increased N fertilization is caused by unbalanced shoot-to-root allocation increasing vegetative growth, reducing nutrient efficiency and inhibiting fruit-set (Albornoz, 2016). For tomatoes, Stefanelli et al. (2010) reported poor fruit set under high-N conditions; and for *Prunus cerasus* (sour cherry), Lindhard and Hansen (1997) reported a reduction in the number of flowers per tree, fruit set, and yield. Optimization of N fertigation (i.e., the process of applying nutrients and water to crops simultaneously) has become a major global concern due to increasing NO_3_^–^ leaching below the root system (Del Amor, 2007; Yasuor et al., 2013). However, the optimization of N application is challenging due to the difficulty of quantifying N uptake by the root system, exacerbated by the opacity and complexity of the root growth environment. Most previous studies that aimed to improve NUE have focused mainly on aboveground plant parts and grain yield as key selection criteria (Zhan and Lynch, 2015). However, the root system plays a crucial role in the uptake of nutrients and water, and the morphology and physiology of the root system changes with the availability of resources in the soil (Grasso et al., 2020; Groenveld et al., 2019). Root traits often exhibit higher heritability than aboveground traits (Pieruschka and Schurr, 2019), indicating the importance of an understanding of root morphology in effective N management. Although the effect of N on root system morphological changes has been demonstrated (Koevoets et al., 2016; Lynch, 2013, 2019), our understanding of how these changes influence N uptake, plant growth and productivity remains limited. This knowledge gap hinders our ability to optimize N applications effectively based on root-system information.

Root traits such as root length, rooting depth, and root length density (RLD) are important parameters in the interaction of plant roots with the environment, and can be used to evaluate the efficiency of plant nutrient and water uptake (Koevoets et al., 2016; Lynch, 2019; Malamy, 2005; Walter et al., 2009). Root-system plasticity allows the adaptation of the root system to environmental stresses and the efficient exploitation of areas rich in resources (e.g., N), thus maximizing nutrient uptake, crop development, and yield (Postma et al., 2014; Smith and De Smet, 2012). For example, a deeper, more spatially distributed root system increases the efficiency of N uptake because NO_3_^–^, the dominant form of N in most agricultural systems, is highly soluble and mobile, and susceptible to leaching below the root zone (Andrews et al., 2013; Lynch, 2013). Vegetables tend to lose N due to their shallow root systems and low NUE (Padilla et al., 2018), while RLD values for tomatoes and peppers increase under lower rates of N application compared with higher rates (Grasso et al., 2020; Lecompte et al., 2008). High rates of N fertigation inhibit root growth and distribution in crops such as wheat (*Triticum aestivum*; Elazab et al., 2016), maize (*Zea mays*; Chun et al., 2005), and cress (*Arabidopsis thaliana*; Zhang et al., 2000).

While the effects of N application on shoot growth and yield in vegetable crops have been extensively studied, root phenotyping remains relatively limited, especially in field conditions, with most studies being undertaken in laboratory settings (Atkinson et al., 2019). The application of rhizotron and minirhizotron (MR) systems has facilitated the *in situ* observation of root traits in field environments (Atkinson et al., 2019), with MR systems being a widely employed non-destructive method for root observation (Mancuso, 2012; Rewald and Ephrath, 2013), allowing repeated measurements of traits such as root length, and root turnover at precise locations (Zeng et al., 2008). Given the importance of N in regulating root system responses, our study aims to explain the physiological mechanisms underlying root growth responses to N availability, emphasizing the role of root plasticity in improving NUE, plant adaptation and productivity under varied N regimes. We hypothesize that reduced N availability enhances root system plasticity, increasing root length, RLD and surface area while balancing nutrient uptake efficiency and shoot development. The study examines the impact of different N regimes on root dynamics and their correlation with shoot development and crop productivity, this will provide insights on resource allocation between the shoot and the root, and how different N affects root growth and distribution.

## 2 Methodology

### 2.1 Experimental site and design

*Capsicum annuum L.* cv. Canon 7158 (Zeraim Gedera–Syngenta, Israel), a commercial hybrid, was used in the experiments.

#### 2.1.1 2022 experimental season

**Figure 1:**
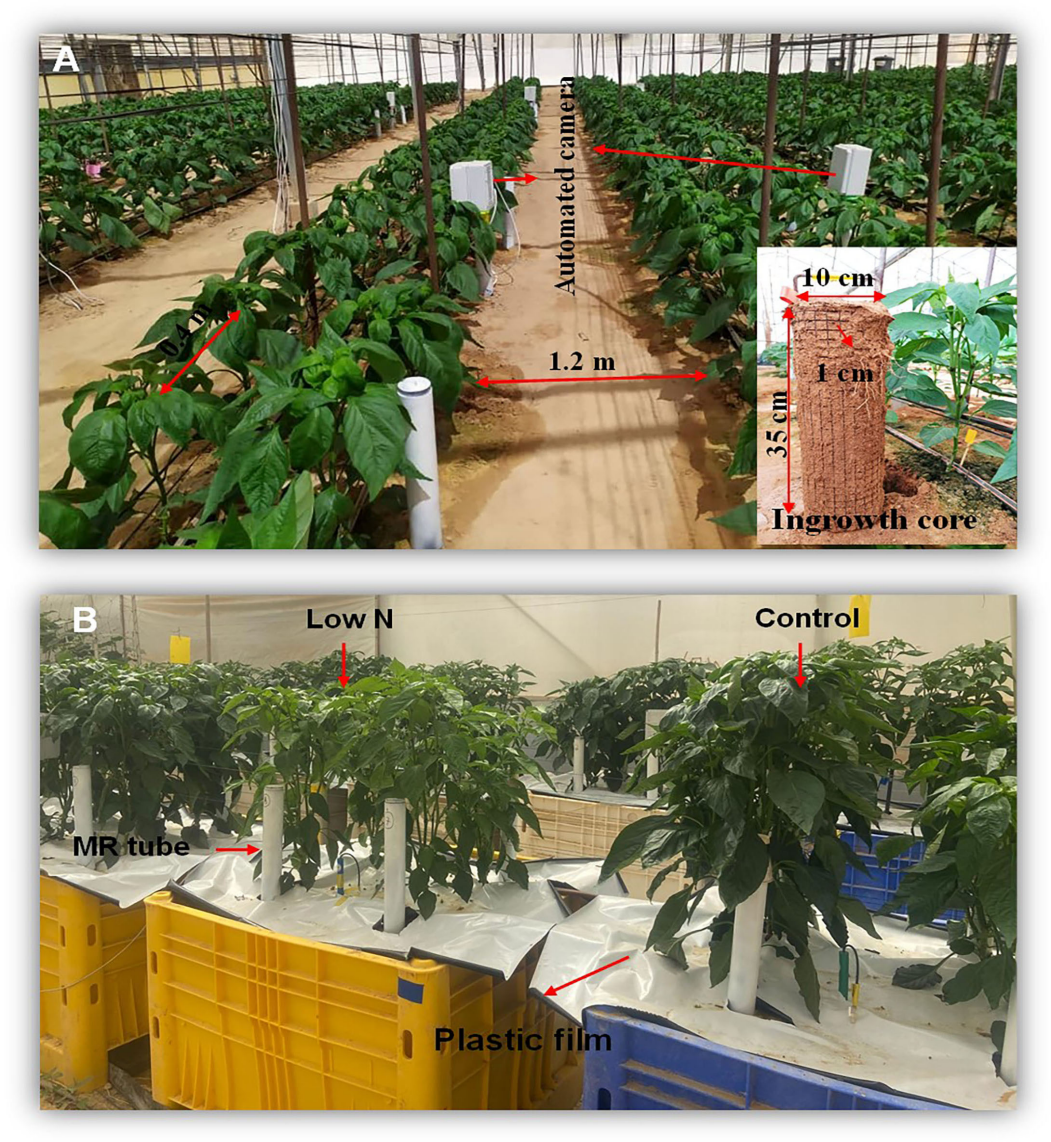
Experimental setup across two seasons. **(A)** 2022 experimental season at 35 days after planting (DAP), showing plant spacing, an automated MR camera, and an ingrowth core for root study. **(B)** 2023 experimental season at 60 DAP, highlighting treatments with low-N (25 ppm) and control (100 ppm), along with the use of minirhizotron (MR) tubes for root observation and plastic film to control evaporation.

The 2022 experiment (**Figure 1A**) was conducted in a net-house on loamy sand soil with sand, silt, and clay contents of 83%, 8%, and 9%, respectively, with a growing area of 750 m^2^ at the Northern and Central Arava R&D Center (30°46′45.3”N, 35°14′31.1”E), Hatseva, Israel from September 2022 to April 2023. During the growing season, the mean temperature varied between 14**°**C and 33**°**C, and the active photosynthetic radiation (PAR) varied between 400 and 1700 µmol m-2 s-1, relative humidity ranged between 20% and 62%, and cumulative precipitation was approximately 55 mm. Photoperiod ranged from 10 h to 13 h, with actual daily sunshine hours varying between 7.5 and 12.4 hours. Two rows of pepper seedlings were planted in soil beds (plots) of 12 m length, with a spacing of 0.4 × 0.4 m and 1.2 m between plots. Fertigation was applied using a pressure-compensated drip irrigation system with a discharge rate of 1.6 L h^–1^ (Netafim Ltd., Hatzerim, Israel) and irrigation amount of 5–6 mm d^–1^ in two irrigation events. The irrigation amount was adjusted according to the growth stage. N–P–K 6:6:6 fertilizer (Haifa Group, Israel) was applied at a concentration of 100 ppm for 45 d after planting (DAP) to allow plant establishment, after which it was changed to N–P–K 7:3:7 until the end of the experiment. This treatment was based on a local practice whereby the P concentration is reduced, and K concentration is increased after transplanting and seedling establishment. N in the N–P–K 7:3:7 was supplied primarily as a mixture of NO_3_^-^ and NH_4_^+^, sourced from ammonium nitrate (NH_4_NO_3_) and potassium nitrate (KNO_3_). The ratio of NO_3_^-^ to NH_4_^+^ was maintained at approximately 4.6:2.4 across all N concentrations (100, 50, and 25 ppm N). This same formulation and ratio were also applied in the 2023 experiment. Three N levels were applied after fruit-set (56 DAP): control N (100 ppm), moderate N (50 ppm), and low N (25 ppm). The changed N concentrations of 50 and 25 ppm were achieved by reducing the 100 ppm N by half and a quarter, while maintaining a constant concentration of P and K by supplying extra sources of P and K (monopotassium phosphate (KH_2_PO4), Potassium sulfate (K_2_SO_4_). Six randomly distributed plots were used for each N level.

#### 2.1.2 2023 experimental season

The 2023 experiment (**Figure 1B**) was conducted in a net-house at the Sede Boqer campus of Ben-Gurion University of the Negev, Israel (30°52′N, 34°47′E), from June–December 2023. During this period, mean temperatures ranged from approximately 14°C to 28°C, relative humidity varied between 20 % and 60 %, and total precipitation was 42 mm. Photoperiod ranged from 10 h to 14 h, with actual daily sunshine hours between 7.5 and 12.6 hours. Ten 500 L containers (100 cm wide, 100 cm long, 50 cm high) were filled with sandy soil (~85% sand, ~10% silt, ~5% clay), with a ‘rockwool’ layer at the bottom. The containers were drilled with the same number and size of holes at the bottom to allow drainage. The surface of each container was covered by white/black plastic film of 22 μm thickness with a white surface on the top (Ginegar Plastics Products Ltd., Kibbutz Ginegar, Israel) to minimize evaporation. Four pepper seedlings were planted in each container, with a spacing of 0.4 × 0.4 m (similar spacing to the 2022 experiment).

Fertigation was applied using a pressure-compensated drip irrigation system with four drippers per container, with a discharge rate of 1.6 L h^–1^ (Netafim Ltd., Hatzerim, Israel) and 6 mm d^–1^ of irrigation with the same irrigation frequency as the 2022 experiment. N–P–K 6:6:6 fertilizer was applied at a concentration of 100 ppm for 30 DAP to allow plant establishment, after which it was changed to N–P–K 7:3:7 until the end of the experiment. At 30 DAP the two treatments were initiated, based on the 2022 experiment, where moderate-N enhanced both root growth and yield. We selected the extreme low-N to observe if we would have any improvement in yield productivity. The treatments include control (100 ppm) and low-N (25 ppm) applications of N concentration. Similar to the 2022 experiment, the changed P and K concentration in the low-N treatment was maintained, supplying extra sources of P and K monopotassium phosphate (KH_2_PO_4_), Potassium sulfate (K_2_SO_4_). Treatments were randomly distributed, with each having five replicates.

### 2.2 Root observation: minirhizotron and destructive root methods

A minirhizotron (MR) system was used for non-destructive root observation in both experiments. MR tubes, 100 cm in length, were installed vertically at a distance of 10 cm from the plant two weeks before transplanting, to allow sufficient contact between tube and soil. The aboveground part of the tube (~40 cm) was painted black to prevent light penetration and coated with white paint to reflect excess light and to avoid heat absorption. An automated MR camera (RootCam^©^; Crystal Vision, Samar, Israel) shown in **Figure 1A**, with a resolution of 2592 × 1944 pixels was used to take images at intervals of 18.75 mm along the tube every two weeks. Sixteen MR tubes were installed for the control and moderate-N treatments in the 2022 experiment, and four tubes per container in the 2023 experiment, with a total of 40 tubes. Root traits analysis of MR images were done using Rootfly software, which allows for manual and semi-automated annotation of roots from the MR images. Since the image resolution and physical dimensions were known, we calibrated one image in Rootfly to set the scale, and this calibration was applied to all subsequent images. Visible roots were manually traced, and the software calculated root length by converting pixel distances into physical measurements (mm) based on the known scale (Zeng et al., 2008).

Destructive analyses involved the measurement of root parameters (root volume, RLD, surface area, and average diameter) and biomass, using different methods each covering distinct soil volumes: ingrowth cores (2749.25 cm^3^), soil cores (294.56 cm^3^ per 15 cm soil depth), and soil excavation (125, 000 cm^3^). The destructive methods were selected because are suitable alternative to MR root study for examining root biomass and to compare the measured parameters to MR system. The 2022 experiment employed an ingrowth core (shown in **Figure 1**) comprising a wire-mesh cylinder 35 cm long by 10 cm wide with a 1 cm mesh. Twenty-four cores were installed two weeks before transplanting (on the same day as MR tube installation) for the stabilization period, 15 cm from the plant. The first ingrowth core sample was collected three months after transplanting and replaced with new ingrowth cores, which were collected at the end of the experiment. For root sampling at the end of the 2023 experiment, two plants were excavated, each from one-quarter of the container (125 L soil). Additionally, two soil cores per container of 5 cm diameter were taken at 15 cm depth intervals (0-15, 15-30, 30-45) 10 cm from the plant.

After sampling, roots were washed using a 0.2 mm sieve and stored at 5°C in 70% ethanol. Scanning was performed using an Epson Expression 10,000XL digital scanner (Epson America, Inc., USA). Root length, diameter, surface area, volume, and RLD were estimated from scanned images using the WinRhizo analysis program (RHIZO Regent Instruments, Quebec, Canada).

### 2.3 Morphological measurements

At the end of each experiment, the aboveground parts of plants were separated into stems, leaves, and fruit, which were weighed fresh before stems and leaves were oven-dried at 65°C in a ventilated oven to constant mass. Yields refer to the cumulative collection of each harvest over the experimental period. The total N applied for the different treatments was calculated for each experiment based on irrigation volume and average N concentration over the growing season. NUE was calculated as an agronomic NUE (ANUE) using Equation 1 based on total marketable yield and total N applied (Yasuor et al., 2013).


(1) $$\text{ANUE}=\frac{\text{Total yield }\left({\text{kg ha}}^{-1}\right)}{\text{Total N applied }\left({\text{kg ha}}^{-1}\right)}$$

### 2.4 Leaf gas exchange

Leaf gas exchange (stomatal conductance rate (g_s_; mol H_2_O m^–2^ s^–1^) and photosynthesis rate (A_n_; μmol CO_2_ m^–2^ s^–1^)) was determined using an infrared gas analyzer (LICOR 6800, LICOR, Lincoln, USA) for the fourth leaf from the top of the stem (i.e., the youngest fully expanded leaf exposed to sunlight). Measurements were performed at a constant photosynthetic active radiation (PAR) of 800 μmol m^–2^ s^–1^, a constant CO_2_ level of 400 μmol mol^–1^, a relative humidity of 25%–30%, and a leaf temperature of 25°C. For each treatment, six biological replicates were measured and two technical replicate per plant. Gas exchange measurements were undertaken between the hours of 11:00 and 14:00 on clear days.

### 2.5 Statistical analysis

Analysis of variance was performed using the John’s Macintosh Project (JMP) statistical package (SAS Institute Inc., Cary, USA). A *post-hoc* Tukey honestly significant difference (HSD) test was performed to determine the statistical significance between treatments in terms of measured parameters. Figures were produced using SigmaPlot (Systat Software, San Jose, USA) and R software (www.r-project.org). Significance was set at *p*< 0.05.

## 3 Results

### 3.1 Effect of N on leaf gas exchange

**Figure 2:**
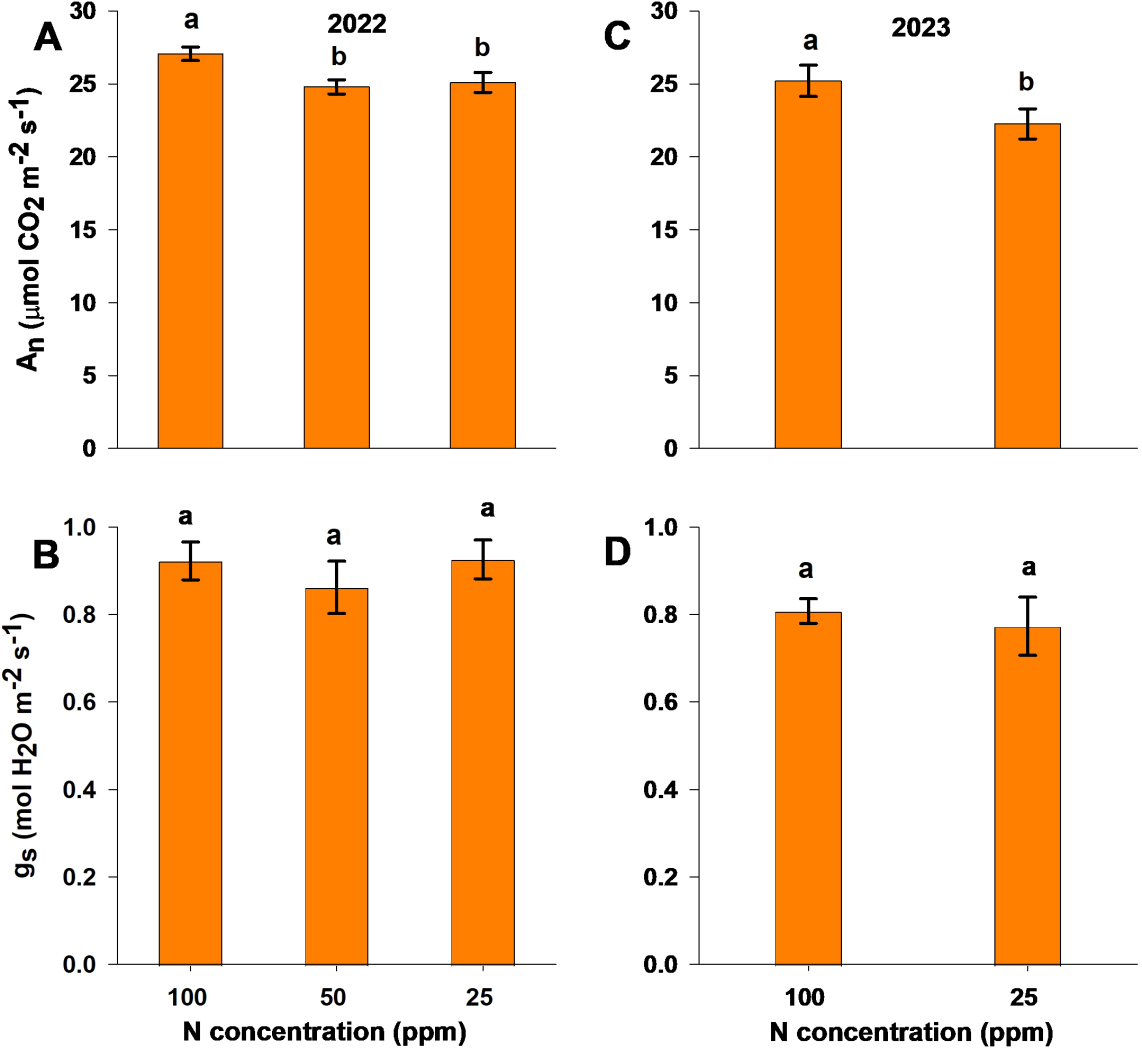
Photosynthetic rate (A_n_; **A, C**) and stomatal conductance (g_s_; **B, D**) under different nitrogen levels in the 2022 **(A, B)** and 2023 **(C, D)** experimental seasons, measured at 90 and 92 days after planting, respectively. Different letters above the bars indicate statistically significant differences between mean values (Tukey’s HSD test, p< 0.05). Error bars represent the standard error; n = 6.

Measurements of leaf gas exchange were undertaken at 90 and 92 DAP for the 2022 and 2023 seasons. For both experiments, A_n_ differed significantly between treatments, with reduced N (50 and 25 ppm) treatment resulting in a lower A_n_ than the control treatment (**Figures 2A, C**). For g_s_, there was no significant difference between treatments in either experiment (**Figures 2B, D**).

### 3.2 Influence of N concentration on fresh yield and dry biomass

**Figure 3:**
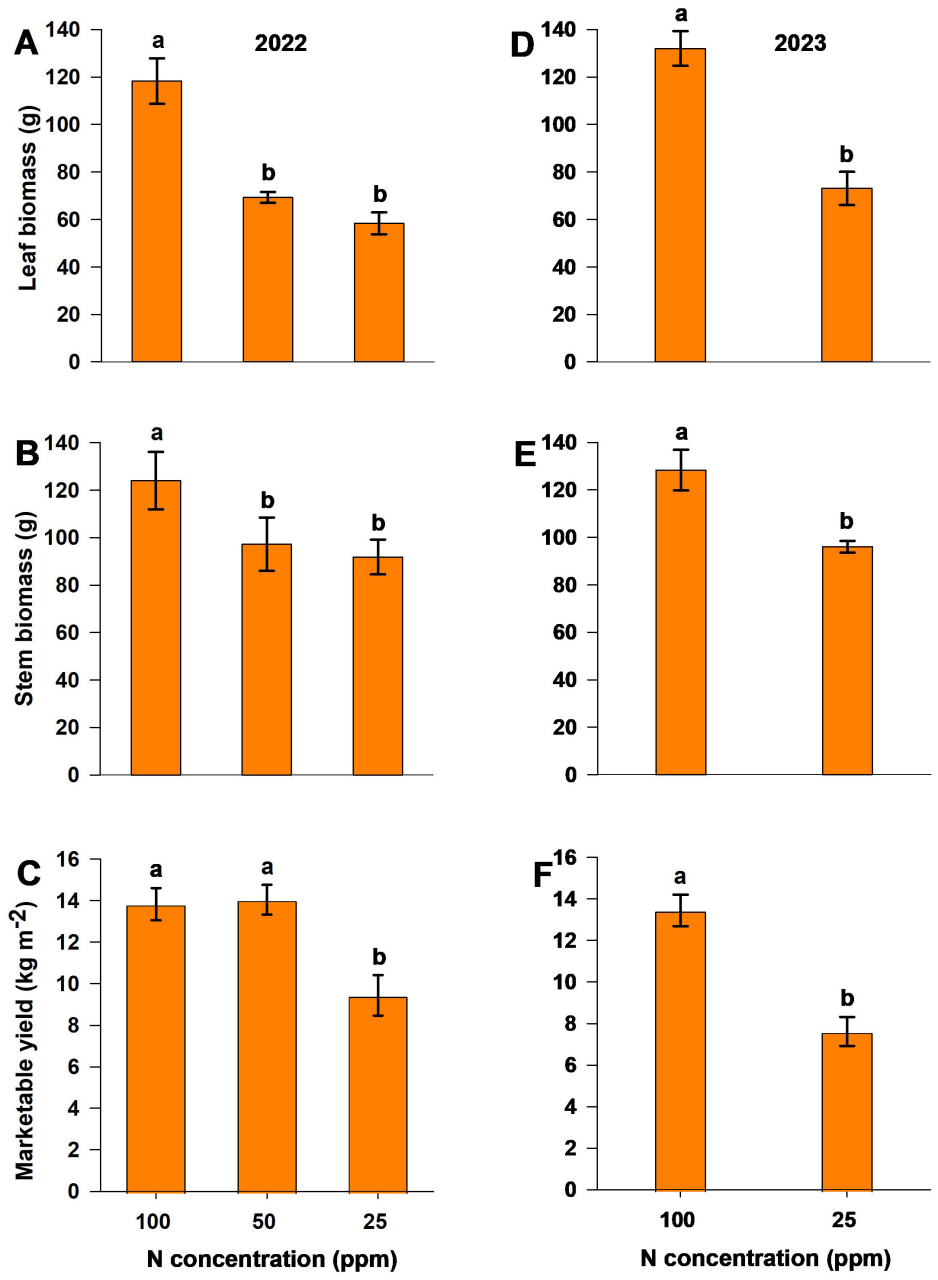
Dry leaf biomass **(A, D)**, dry stem biomass **(B, E)**, and fresh marketable yield **(C, F)** for different nitrogen levels in the 2022 **(A–C)** and 2023 **(D–F)** experimental seasons. Different letters above the bars indicate statistically significant differences between mean values (Tukey’s HSD test, p< 0.05). Error bars represent the standard error; n = 6.

The dry biomass of leaves and stems was measured at the end of both experiments. A reduction in N (the moderate-N and low-N treatments) resulted in a significant reduction in biomass production (**Figures 3A, B, D, E**). Marketable yield, recorded as the cumulative harvest throughout the experiment, was significantly lower with the low-N (25 ppm) treatment compared to control and moderate-N treatments in both experimental years. In the 2022 season, the moderate-N (50 ppm) treatment resulted in yields of 2% and 18.5% higher than those of the control (100 ppm) and low-N treatments, respectively (**Figures 3C, F**). However, there was no significant difference between the yields of the moderate-N and control treatments (**Figure 3C**). Marketable yield responded positively to the moderate-N treatment relative to the low-N and control treatments, with the latter resulting in no significant increase in yield.

### 3.3 Root growth response to different N levels (MR system)

**Figure 4:**
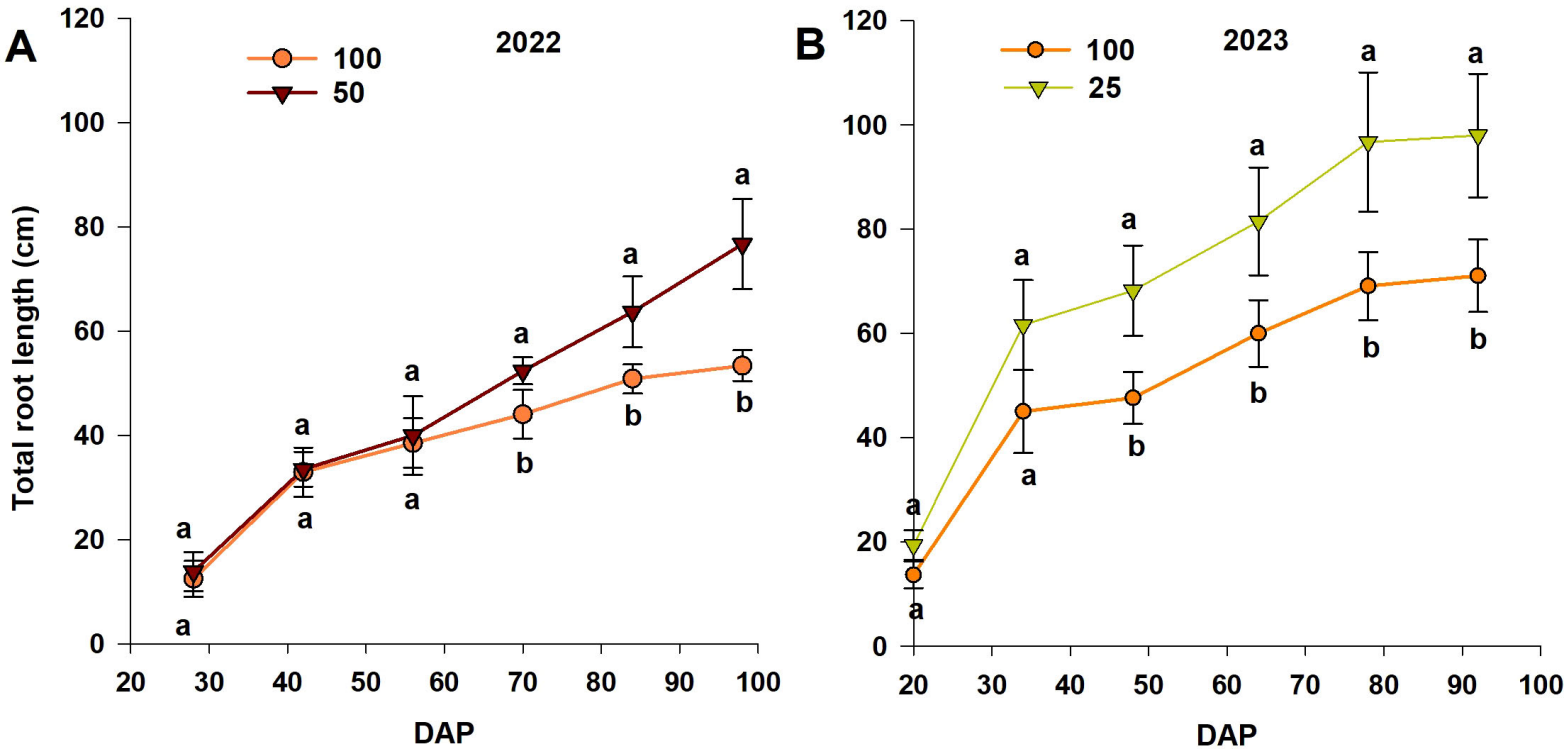
Trends in total root length (from the MR system) with different nitrogen levels in **(A)** 2022 and **(B)** 2023 (DAP = days after planting). Different letters above the bars indicate statistically significant differences between mean values (Tukey’s HSD test, p< 0.05). Error bars represent the standard error; n = 6.

The total root length increased linearly with time during both experimental seasons, for each treatment (**Figure 4**). There was no significant difference between treatments before the initiation of treatment and at 56 and 34 DAP after treatment initiation in 2022 and 2023, respectively. Significant differences between treatments were observed from 70 DAP (~14 days after the start of treatment) until the end of measurements (98 DAP), with root length increasing in the moderate-N case (**Figure 4A**). For the 2023 experiment, root length increased significantly with the low-N treatment, relative to the control treatment at 18 d after the start of treatment (**Figure 4B**). Trends in the effect of the reduced-N treatment were similar for both experiments, with the low-N treatment resulting in a greater root length than the control and moderate-N treatments (**Figure 4**).

**Figure 5:**
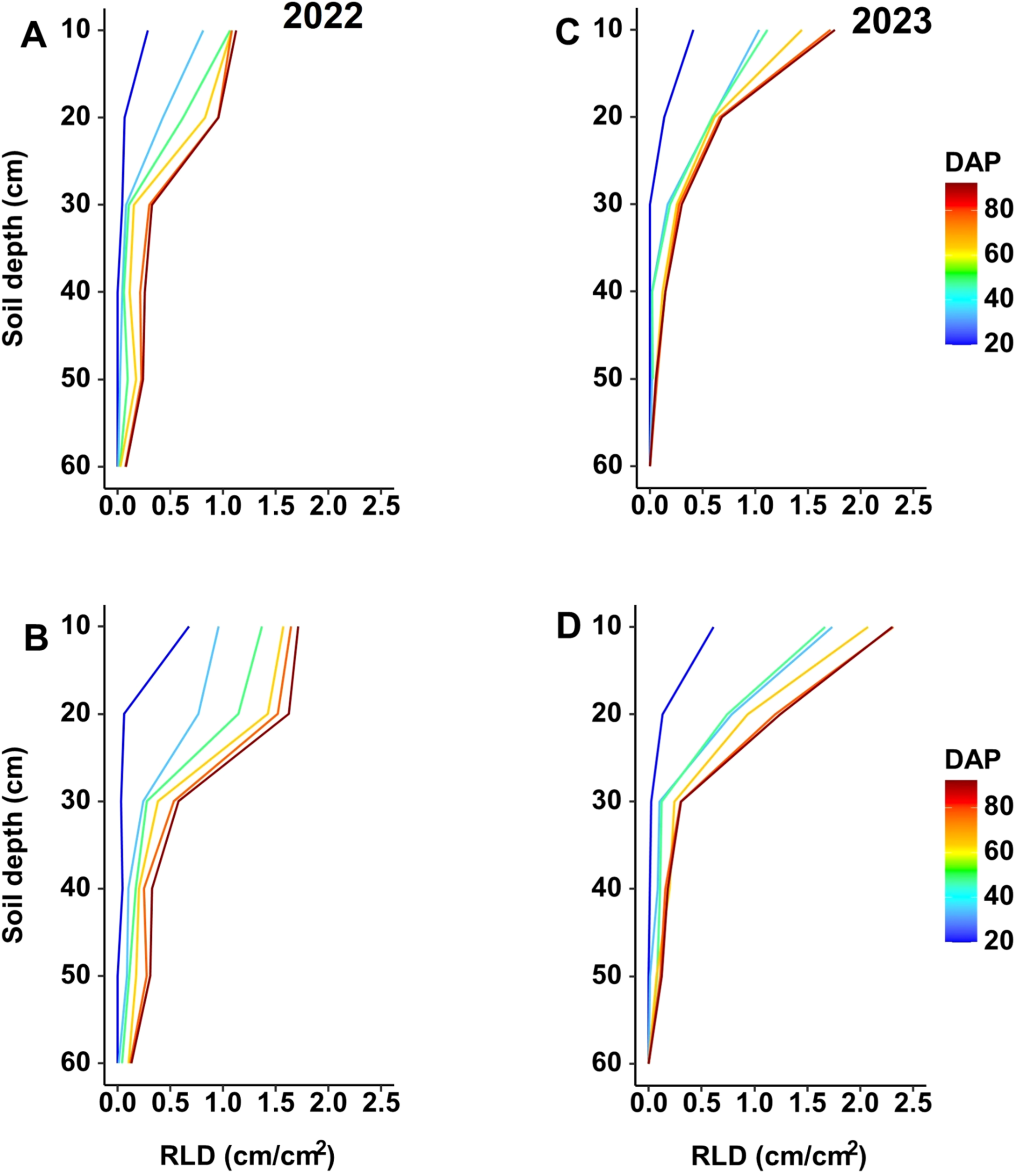
Changes in RLD with depth over time with different N treatments (**(A, C)** 100 ppm; **(B)** 50 ppm; and **(D)** 25 ppm) during the two experimental seasons. Measurements were taken on different days after planting (DAP) during the **(A, B)** 2022 and **(C, D)** 2023 seasons.

The RLD changes with depth over time (**Figure 5**) for both experimental seasons indicate higher values in the upper 30 cm of the soil profile. In the 2022 season, the moderate-N treatment yielded a significant increase in RLD in the upper 30 cm relative to the control treatment (**Figures 5A, B**), while in 2023 the low-N treatment yielded a significantly higher RLD compared to control (**Figures 5C, D**). The same trend was obtained for both seasons, with an increase in RLD under reduced-N treatment and with higher RLD values in the upper 30 cm of the soil profile, and with the low-N treatment yielding a value that was ~0.8 cm cm^–2^ higher than the value in the moderate-N treatment (**Figure 5**).

### 3.4 Root growth response to different N levels (Destructive root study method)

**Figure 6:**
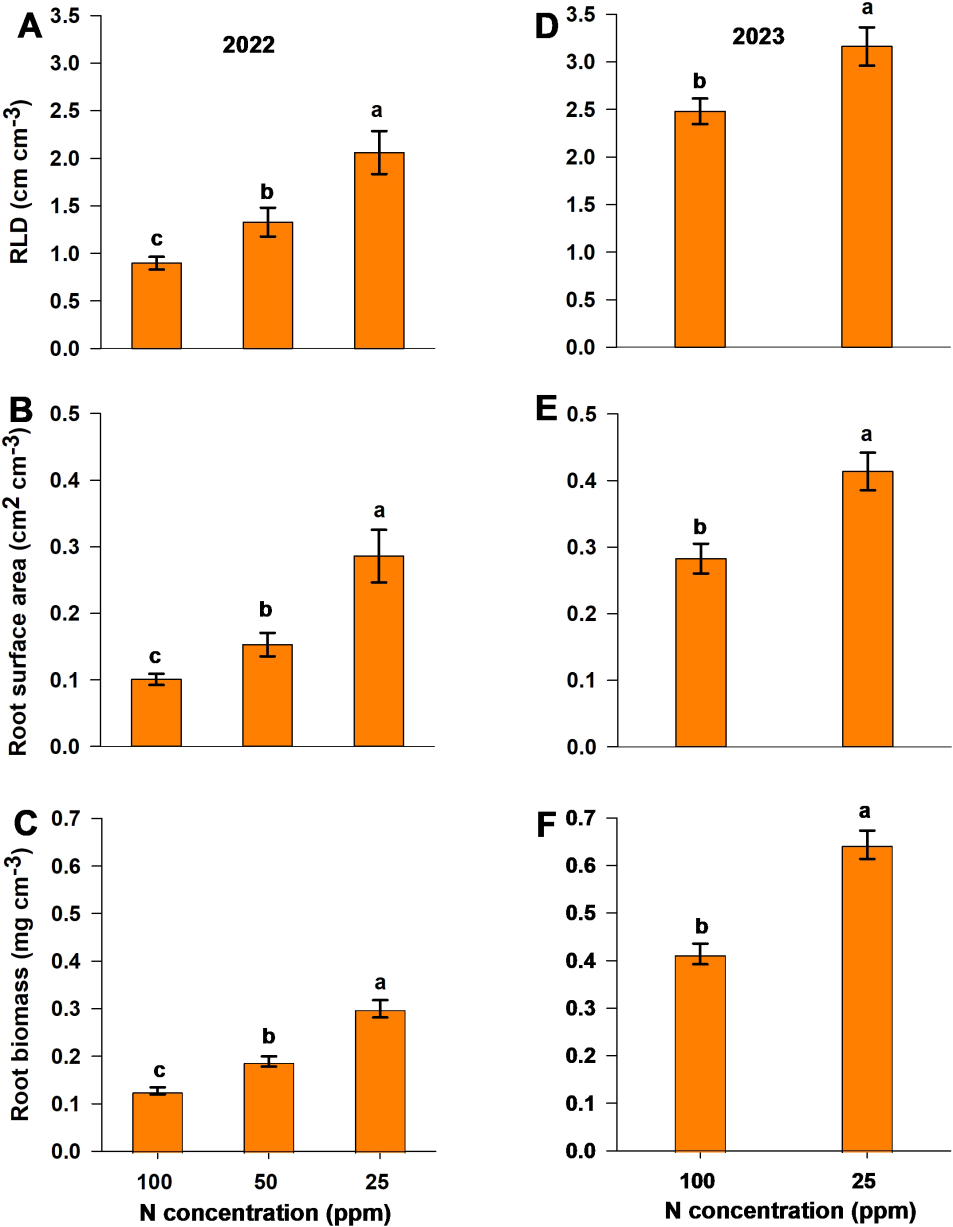
Root parameters measured using **(A–C)** the ingrowth core method and **(D–F)** the soil excavation method in response to different N levels during the two seasons. The panels show **(A, D)** RLD, **(B, E)** surface area, and **(C, F)** root biomass values per unit soil volume in the **(A–C)** 2022 and **(D–F)** 2023 experimental seasons. Different letters above the bars indicate statistically significant differences between mean values (Tukey’s HSD test, p< 0.05). Error bars represent the standard error; n = 6.

RLD, root surface area, and root biomass values per soil volume for both experiments were measured using the ingrowth core method for the 2022 season and the soil excavation method for the 2023 season. Both methods yielded significantly higher values for each parameter (RLD, root surface area per soil volume, and root biomass per soil volume) with the low-N treatment compared to moderate-N and control (**Figure 6**). Additionally, moderate-N treatment showed a significant increase in the above measured parameters compared to control in the 2022 season. Soil excavation data indicate higher values for each parameter relative to ingrowth-core data (**Figures 6D–F**). Overall, the low-N and moderate-N treatments resulted in a significant increase in all root parameters relative to the control treatment, for both seasons.

### 3.5 Root length density distribution under root diameter

**Figure 7:**
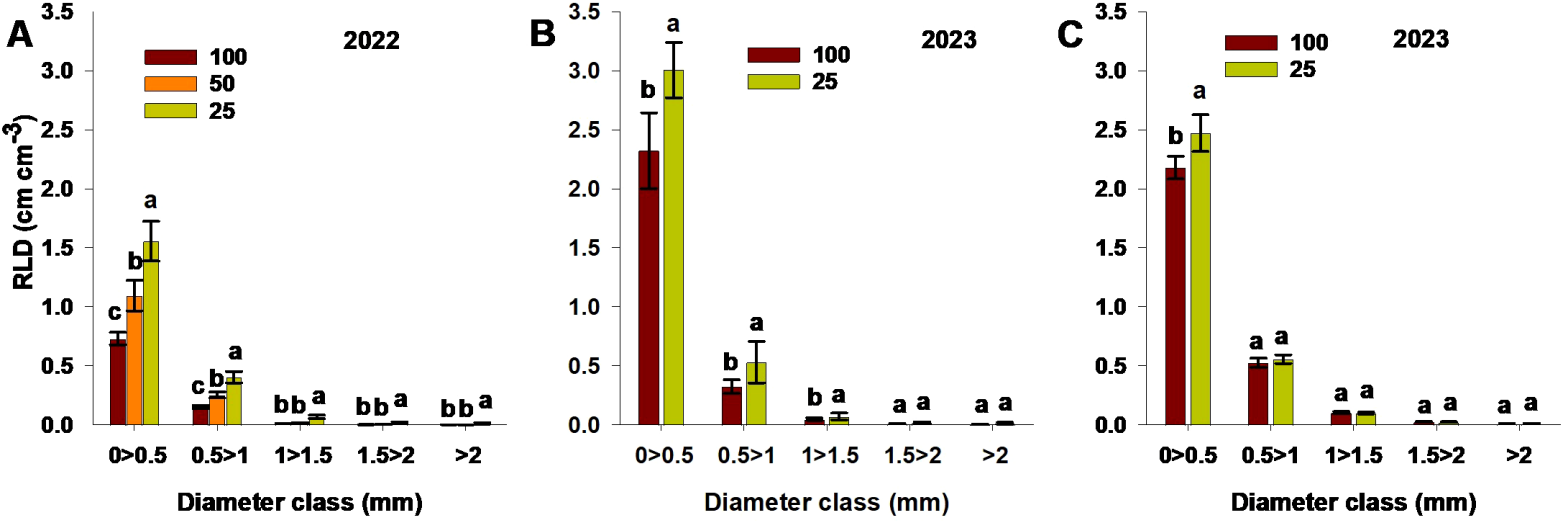
RLD values for different root diameter ranges, measured using **(A)** the ingrowth core method, **(B)** the soil core method, and **(C)** soil excavation, for different N treatments (100; control,50; moderate-N 25; low-N) in the **(A)** 2022 and **(B, C)** 2023 seasons. Different letters above the bars indicate statistically significant differences between mean values (Tukey’s HSD test, p< 0.05). Error bars represent the standard error; n = 6.

RLD values were distinguished by root diameter for both experimental seasons and for each destructive study method (ingrowth core, soil core—combined from the three soil depths, and soil excavation) (**Figure 7**). For all study methods, roots of ≤0.5 mm diameter had the highest RLD values. The low-N treatment resulted in significantly increased RLD values across all diameter classes relative to the moderate-N and control treatments, using the ingrowth core method. The low-N treatment also resulted in significantly increased values for roots of<1.5 mm diameter relative to the control treatment, using both the soil core and soil excavation methods (**Figure 7**). Overall, the control treatment limited RLD values across all diameter classes for all methods, while the low-N treatment resulted in significantly higher RLD values compared to moderate-N and control.

### 3.6 Principal component analysis

**Figure 8:**
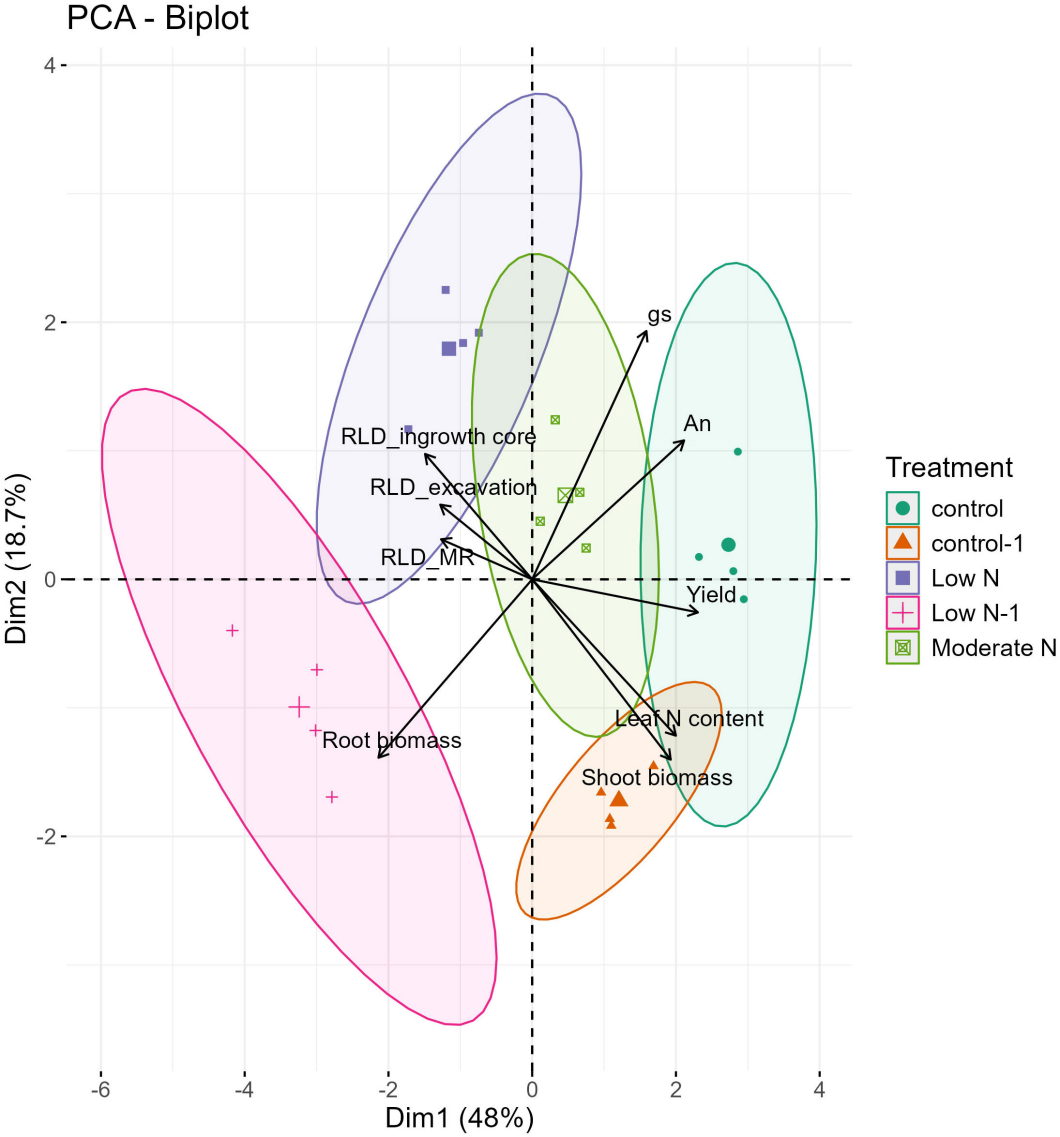
Biplot of the principal component analysis related to the above-ground measured parameters [Shoot biomass, yield, photosynthesis rate (An), Stomatal conductance rate (gs), and leaf N content] and root length density (RLD) under the three root methods used [RLD_MR (data point at 30 cm soil profile), RLD_ingrowth core, and RLD_excavation]. Each treatment is represented by a different symbol. Control (green circles, 2022), Moderate N (light green squares, 2022), Low N (blue squares, 2022), Control-1 (orange triangles, 2023), and Low N-1 (pink crosses, 2023).

PCA conducted on several parameters, including RLD measured using different methods and above-ground morphological and physiological traits, captured 66.7 % of the variability on the first two principal components. PC1 accounted for 48 % of the total variation, and PC2, 18.7 % (**Figure 8**).

Treatments under Low N and Low N-1 clustered to the left along PC1 and were associated with enhanced root biomass and RLD. In contrast, control and control-1 treatments were positioned to the right along PC1, which was strongly associated with above-ground traits such as leaf N content, g_s_, A_n,_ and yield. This positioning indicates that higher N availability favored shoot-related physiological and morphological traits, while reduced N enhanced below-ground growth and development. Notably, moderate-N treatment was positioned in the center, correlating strongly with higher yield and root traits, reflecting a balanced resource allocation between root and shoot systems.

## 4 Discussion

In the present study, higher N application rates enhanced aboveground morphological and physiological parameters, while lower application rates reduced such morpho-physiological parameters (**Figures 2**, **3**). Approximately 75% of total leaf N is located within the photosynthetic system (Shangguan et al., 2000); consequently, with the low-N application, the A_n_ rate was significantly reduced due to insufficient leaf N (**Figure 2**). This interpretation is supported by measured leaf N content and petiole NO_3_^-^ sap concentration, which reduced significantly under low N availability (**Supplementary Figure 4**). This finding is consistent with that of Souri and Dehnavard (2018), who reported reduced photosynthesis and stomatal conductance with N deficiency in tomatoes. However, in our experiments, the moderate-N and low-N treatments did not negatively affect stomatal conductance, suggesting that the reduction in photosynthesis under reduced-N conditions was driven mainly by biochemical limitations such as reduced chlorophyll production, enzyme activity, and protein synthesis, rather than changes in stomatal behavior (Sun et al., 2023).

Root growth observations from the MR system and destructive methods under different N application rates revealed trends opposite to those recorded in aboveground morphological and physiological performance (**Figure 8**). While reduced N application reduced aboveground growth, it promoted root growth and distribution, consistent with previous reports (Lynch, 2019; Zhan and Lynch, 2015). The present results show an increase in all root traits (root length, RLD, and root surface area) under moderate-N and low-N treatments, as measured by both the MR system and destructive methods, for both experiments (**Figures 4**–**6**). This is consistent with the studies of Zhang et al. (2000) and Fernandez et al. (2020), who reported inhibited root growth with higher N levels, and increased growth with reduced N conditions for *Arabidopsis thaliana* and cotton, respectively. The increase in measured root traits can be explained by the enhanced development of primary and lateral roots under reduced N conditions, with the root system exploring deeper and larger soil layers for available N (Koevoets et al., 2016; Lynch, 1995).

RLD changes with soil depth, recorded by the MR system, indicated significantly higher RLD values in the upper 30 cm of the soil profile under the moderate-N and low-N treatments relative to the control treatment (**Figure 5**), with no increase in RLD in deeper soil layers. This result is consistent with the findings of previous studies that N fertilization influences RLD more than rooting depth for sweet pepper (Grasso et al., 2020; Kong et al., 2012), tomato (Lecompte et al., 2008), and cotton (Chen et al., 2018). In the present study, the lack of a significant increase in RLD below 30 cm soil depth indicates the need to minimize the amount of N applied to reduce NO_3_^–^ leaching below the inactive zone of the root system, thus reducing the environmental impact. This finding is further supported by soil core method data, which revealed significant differences in root length, RLD, and root surface area among treatments within the top 30 cm of the soil profile, while no differences were observed at the 30–45 cm soil depth (**Supplementary Figure 1**). Furthermore, our results indicate a significant increase in TRL with the low-N and moderate-N treatments, particularly after treatment initiation, with an increase in RLD reflecting an adaptive strategy of the plant in optimizing nutrient uptake over time. This enhanced root development and distribution under reduced N levels suggests that optimized N applications not only conserve resources but also improve root system functionality, ultimately enhancing NUE, as indicated by the higher ANUE recorded under reduced-N conditions in both experiments (**Table 1**). Similarly, Guo et al. (2024) reported that under moderate N application (225 kg ha^-1^), root developed a longer, thinner, and deeper profile, which enhanced root efficiency for water and N uptake compared to higher N application (300 kg ha^-1^).

**Table 1:** Nitrogen concentration (ppm), total applied N (kg ha^–1^), and agronomic nitrogen use efficiency (ANUE) for each treatment for the 2022 and 2023 experiments.

Year	N treatment	NO_3_-N	NH_4_-N	Irrigation N concentration (ppm)	Total N applied (kg ha^–1^)	ANUE
2022	Control	65.7	34.3	100	973	71.12^c^
	Moderate N	32.8	17.2	50	630	111.43^b^
	Low N	16.4	8.6	25	469	122^a^
2023	Control	65.7	34.3	100	1018	70.92^b^
	Low N	16.4	8.6	25	468	102.78^a^

Different letters indicate statistically significant differences (Tukey’s HSD test, P< 0.05) between treatments within each year.

P and K were supplied at a constant concentration of 23 and 100 ppm, respectively.

The destructive methods of root analysis indicate a significant increase in RLD under reduced-N conditions, consistent with observations of the MR system. This consistency is further supported in **Figure 8**, where RLD from MR system, ingrowth cores, and excavation clustered closely in the same region, indicating that all three methods captured similar treatment-related patterns in root development. Similar findings have been reported for wheat (Rasmussen et al., 2015) and pepper (Hu et al., 2021; Padilla et al., 2017). However, RLD measured with the MR system was greater than that observed with the destructive root methods. This may be attributable to factors such as the loss of fine roots during washing, or roots occupying a greater soil volume. Similar observations have been reported in studies of cotton (Chen et al., 2018), which recorded higher RLD values using the MR system than in soil core measurements. However, contradictory findings were reported by Samson and Sinclair (1994), who observed lower RLD values in the upper 30 cm of the soil profile with the MR system relative to the ingrowth core method. Differences in RLD values across the three destructive methods may be attributed to factors such as the timing of sampling and the excavated soil volume. Differences across all root study methods indicate that the choice of method may affect the results; consequently, the use of multiple methods together may provide more accurate data.

The increase in root surface area with the moderate-N and low-N treatments further highlights the role of reduced N levels in enhancing the spatial distribution of roots, and increasing soil exploration and nutrient uptake efficiency (Lecompte et al., 2008; Mahgoub et al., 2017).

Fine roots (<2 mm diameter) are crucial for water and nutrient absorption, moderate-N and low-N treatments exhibited increased RLD values (**Figure 7**) and root length (**Supplementary Figure 2**). The distribution of such fine roots within specific soil locations serves as an indicator of how resources are distributed in the soil (Kong et al., 2017; Sierra Cornejo et al., 2020). Here, the increased RLD and root length was accompanied by a corresponding increase in root surface area, serving as a compensatory mechanism to enhance nutrient acquisition. Reduced N availability in the soil thus stimulates root growth and distribution, increasing the root surface area for nutrient acquisition without a corresponding increase in carbon allocation to the root, as highlighted by Marschner (2011). A decrease in RLD and root length of fine roots (<2 mm diameter) has been reported with increased N application (Liang et al., 2023) and increased root surface area under reduced N conditions for cotton (Fernandez et al., 2020). However, Wang et al. (2019) reported a contrary observation, where root surface area increased with increased N application, likely due to the combined effects of N and irrigation. Such discrepancies indicate how root response to N may be influenced by other environmental factors.

Enhanced root growth under reduced-N conditions led to an increase in root biomass (opposite that observed for shoot biomass), consistent with observations of sweet pepper (Grasso et al., 2020), where a low N application rate (88 kg ha^–1^) yielded an increase in root growth and biomass. In contrast, increased N levels enhance vegetative growth and reduce root growth (Garnett et al., 2009; Stefanelli et al., 2010; Yasuor et al., 2013), as recorded here (**Figures 3**, **6C, F**). The increase in root biomass relative to shoot biomass led to a significant increase in root/shoot ratio under low-N conditions of ~0.18 higher than the control level (**Supplementary Figure 3**) This allocation pattern reflects the adaptive response of plants to varying N conditions: under moderate-N and low-N levels, resources are redirected to the growth and development of belowground parts to optimize nutrient acquisition, while under high-N conditions, resources are allocated towards aboveground growth. However, while root growth may be significantly enhanced by the low-N treatment, yield and dry-matter production were significantly reduced (**Figures 3**, **6**), indicating that an increase in root growth with low-N treatment may not be sufficient to achieve high productivity. In contrast, the moderate-N treatment demonstrated a more balanced allocation between root, shoot growth, and yield, as illustrated in **Figure 8**. This also demonstrates the adaptive growth of root morphology when N supply is limited (Lynch, 1995, 2013).

ANUE values increased significantly with a reduction in N application (**Table 1**), indicating increased efficiency with reduced N levels, consistent with other studies of peppers (Rodríguez et al., 2020; Yasuor et al., 2013). However, with significantly higher ANUE under low-N treatment, yield was significantly reduced. This suggests that a higher ANUE may be insufficient to offset the effects of low N availability, resulting in reduced yield (Rodríguez et al., 2020). On the other hand, the moderate-N treatment resulted in a higher ANUE than that of the control treatment, but with a similar yield. Despite the reduction in yield under the low-N treatment (~2 kg m^–2^ lower than the moderate-N and control treatments), the available N was sufficient to significantly increase root growth and probably enhance nutrient uptake, resulting in a lesser difference in yield productivity.

Overall, our results indicate that by reducing N application, a balance can be achieved between maintaining productivity and reducing N input. This balance appears to be supported by enhanced root growth and distribution, which are commonly associated with improved nitrogen acquisition. This approach thus promotes sustainable agriculture and mitigates the environmental impact of excessive N use. Future work should include further reduced N treatments, with N concentrations of 25–50 ppm and<25 ppm, for the study of the effect of such levels on root growth and productivity. It may be expected that such an extreme N deficiency would significantly reduce root growth and yield, contrasting with the observations made with the low-N treatment here. However, N fertigation levels of 25–50 ppm might still enhance yield, as implied by the slight increase in yield at 50 ppm relative to the control.

## 5 Conclusion

Our results demonstrate a clear balance and shift in resource allocation between aboveground and belowground plant components under varying N levels. Reduced N application led to increased root growth, as observed in traits such as TRL, RLD, and surface area by both destructive and non-destructive root analysis methods, demonstrating the capacity of bell pepper to optimize nutrient uptake under limited N conditions. Root distributions were greater in the upper 30 cm of the soil profile for all the treatments but significantly greater with the moderate and low-N treatments, indicating that bell pepper roots are concentrated in the upper 30 cm of the soil profile, and with minimization of N application thus being necessary to reduce NO_3_^–^ leaching below the inactive root zone. While moderate-N enhanced root growth and productivity, low-N significantly enhanced root growth and NUE without sufficient yield improvement. These results indicate that optimizing N fertigation can improve root growth and distribution, enhancing NUE while maintaining productivity, providing a sustainable approach to fertilization management in bell pepper cultivation.

## Data Availability

The raw data supporting the conclusions of this article will be made available by the authors, without undue reservation.
