# Supplementary material for: Root growth and yield responses to nitrogen levels in bell pepper (Capsicum annuum) cultivation: balancing nutrient efficiency and productivity
Source: Front Plant Sci. 2025 Sep 8;16:1589560. doi: 10.3389/fpls.2025.1589560 (PMC12456085; doi:10.3389/fpls.2025.1589560)
Supplement: Supplementary file 1 [file DataSheet1.docx]

Supplementary Material


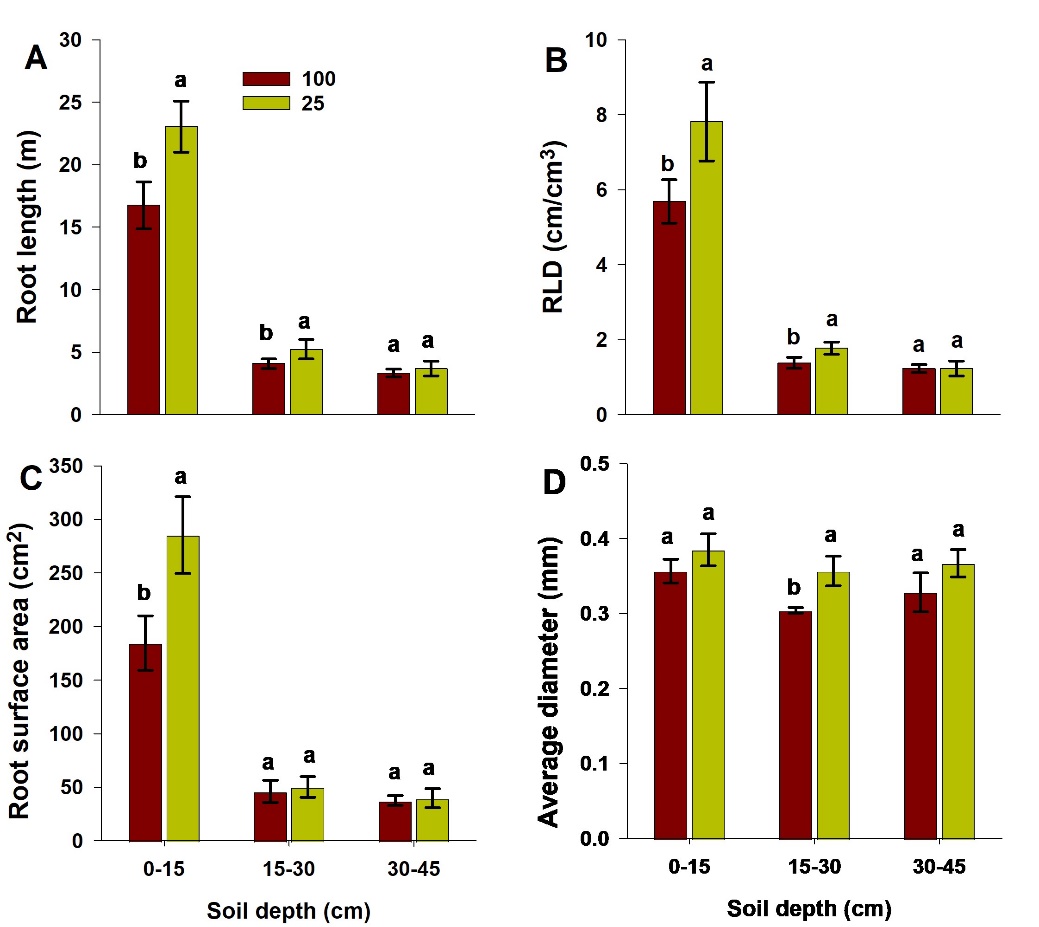


**Supplementary Fig. 1.** Root parameters from soil core method (2023 experiment) at different soil depths under different nitrogen levels (100; control, 25; Low-N). **(A)** root length, **(B)** RLD, **(C)** surface area, and **(D)** average diameter. Different letters above the bars indicate significant differences between means (Tukey’s HSD test, 𝑃<0.05). Error bars represent the standard error. n=6.


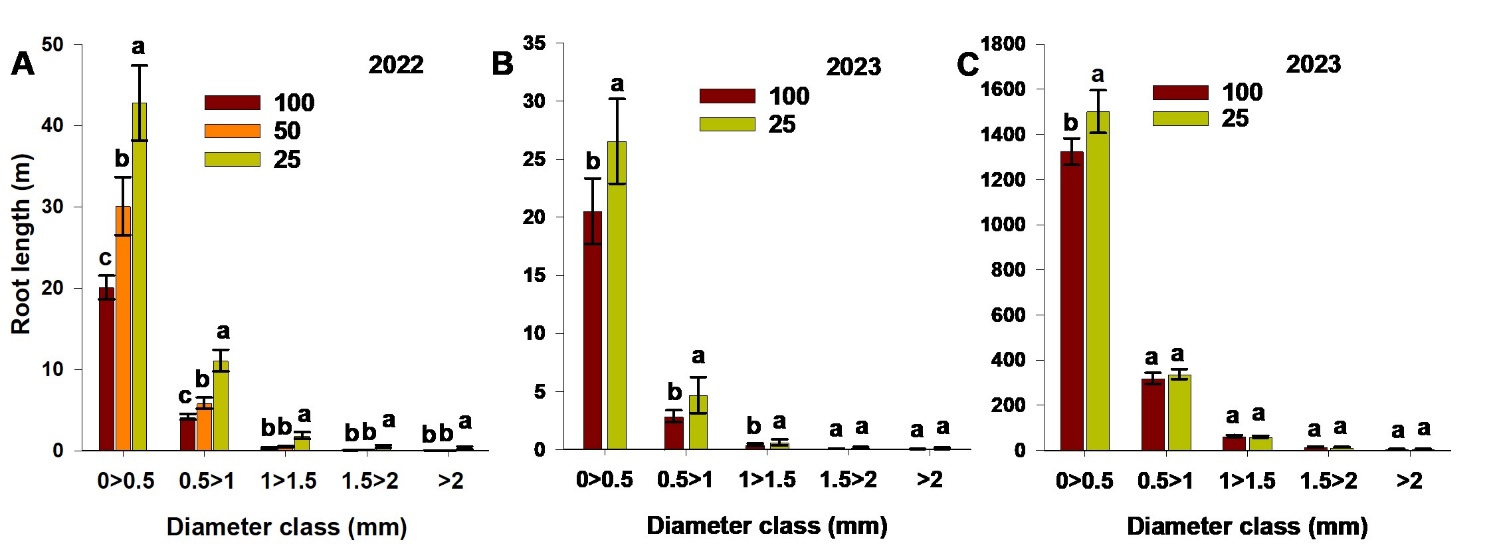


**Supplementary Fig. 2.** Distribution of root length under different root diameter ranges, measured using **(A)** the ingrowth core (0.00275 m^3^), **(B)** the soil core (0.00087 m^3^), and **(C)** soil excavation (0.125 m^3^), for different N treatments (100; control, 50; moderate-N, 25; low-N) in the **(A)** 2022 and **(B, C)** 2023 seasons. Different letters above the bars indicate statistically significant differences between mean values (Tukey’s HSD test, p < 0.05). Error bars represent the standard error; n = 6.


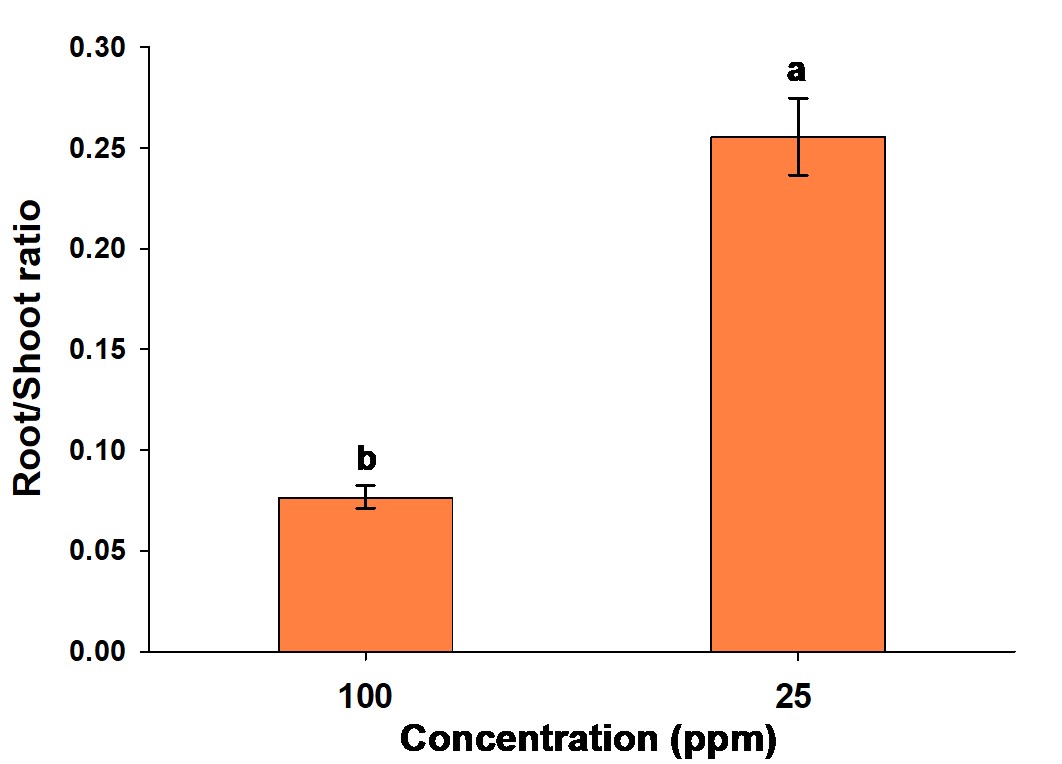


Supplementary Fig. 3. Root-shoot ratio response to different nitrogen levels in the 2023 experiment calculated from the soil excavation method. Different letters above the bars indicate statistically significant differences between mean values (Tukey’s HSD test, p < 0.05). Error bars represent the standard error; n = 6.


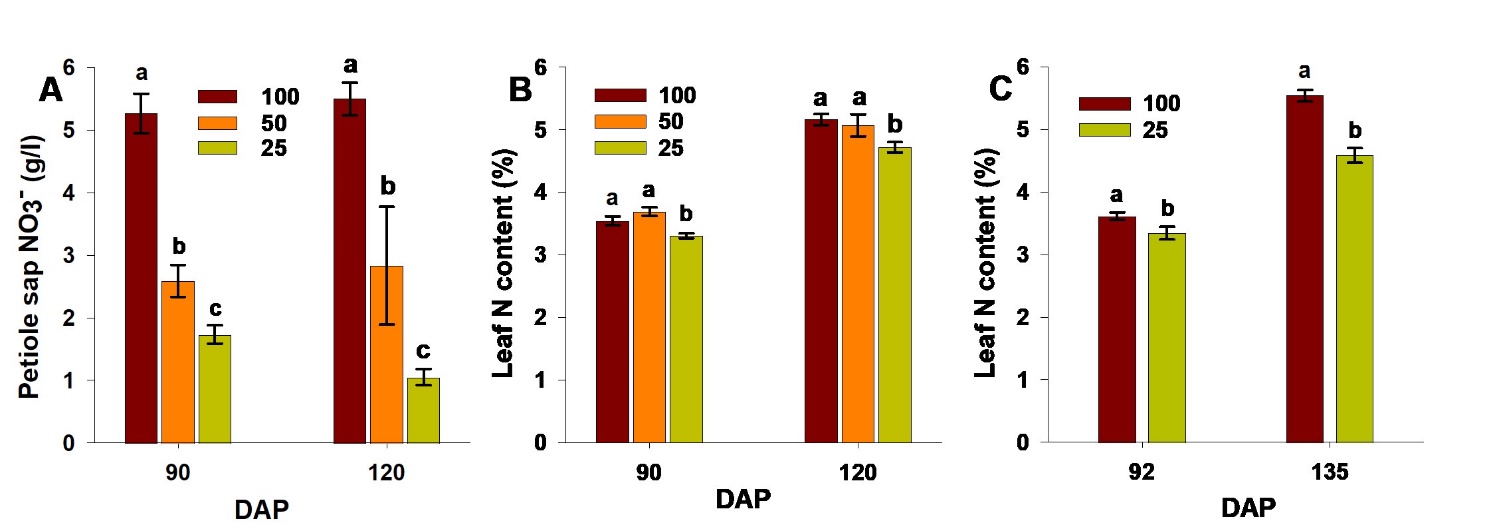


Supplementary Fig. 4. Petiole sap nitrate concentration (A), leaf N content (B and C) response to different nitrogen levels (100; control, 50; moderate-N, 25; low-N) at DAP. (A and B) the 2022 and (B) the 2023 experiment seasons. Different letters above the bars indicate statistically significant differences between mean values (Tukey’s HSD test, p < 0.05). Error bars represent the standard error; n = 6.
